# Combined transcriptomic and metabolomic analysis of phenylpropanoid biosynthesis in the mechanism of leaf angle formation in Sorghum

**DOI:** 10.3389/fpls.2025.1665475

**Published:** 2025-11-10

**Authors:** Jinhong Li, Yiwei Wang, Yuche Zhao, Yanpeng Zhang, Kuangzheng Qu, Zhenxing Zhu, Chunyu Wang, Zhenjun Li, Ling Cong, Shuang Gang, Xiaochun Lu

**Affiliations:** 1 Sorghum Research Institute, Liaoning Academy of Agricultural Sciences, Shenyang, China; 2 Seed Industry Innovation Research Insititute, Liaoning Academy of Agricultural Sciences, Shenyang, China; 3 School of Life Science and Engineering, Shenyang University, Shenyang, China; 4 Internation Black Soil Carbon and Sustainable Land Management Research Centre, Shenyang University, Shenyang, China

**Keywords:** sorghum bicolor, leaf angle, transcriptomics, metabonomics, phenylpropanoid biosynthesis

## Abstract

Leaf angle is a crucial morphological trait for improving crop architecture and facilitating high-density planting. This study aims to explore the mechanism underlying leaf angle formation in sorghum. We used the *el1* mutant, generated through ethyl methane sulfonate mutagenesis in our laboratory, to conduct a comprehensive analysis, including phenotypic, cytological, and integrated transcriptomic and metabolomic studies. At the S3 stage, *el1* leaves exhibited shrinkage, and their leaf angles were significantly smaller compared to those of the wild type (WT). Cytological analyses revealed that at the S1 stage, the auricles of *el1* had larger cell sizes and fewer cells than those of the WT. Metabolomic analysis based on the Kyoto Encyclopedia of Genes and Genomes (KEGG) identified 19 significantly differentiated metabolites, with 10 upregulated and 9 downregulated. Transcriptomics KEGG analysis revealed 858 upregulated and 533 downregulated differentially expressed genes (DEGs). Integrated analysis highlighted that 12 DEGs were associated with trans-5-O-(p-coumaroyl)shikimate in phenylpropanoid biosynthesis, with 11 positively correlated and one negatively correlated DEG. Additionally, 43 DEGs were linked to coniferyl alcohol, with 35 positively correlated and 8 negatively correlated in *el1* compared to WT. This study establishes a theoretical foundation for understanding the molecular mechanisms by which phenylpropanoid biosynthesis influences leaf angle formation in sorghum and offers a basis for optimizing plant architecture to enable high-density planting.

## 1 Introduction

Sorghum (*Sorghum bicolor*) is one of the major cereal and dryland crops in China. Its yield is closely linked to several industries, including winemaking (Gao et al., 2024; Zhang et al., 2013; Zou et al., 2020), food consumption (Yonemaru et al., 2009), livestock feed production, and bioethanol production (Zhang et al., 2010). In recent years, the available cultivated land in China has decreased, and the sorghum planting area has experienced a slight decline. Nevertheless, the demand for sorghum has increased due to intensified agricultural development. Thus, increasing yield per unit area represents a sustainable strategy to enhance total production on limited cultivated land.

Leaf angle is a vital visual morphological trait for improving crop plant architecture and serves as a critical agronomic indicator for assessing the suitability of elite varieties for high-density planting (Gang et al., 2023; Guo et al., 2021; Moldenhauer and Gibbons, 2003; Zhou et al., 2017). An appropriate leaf angle allows for the rational distribution of received light energy across each leaf layer in high-density planting (Liu et al., 2022). This distribution maintains a relatively high light transmittance within the population, reducing self-shading and minimizing competition from neighboring plants. Consequently, it improves light interception and enhances photosynthetic efficiency (Cao et al., 2022; Liu et al., 2021a; Jiao et al., 2010; Sun et al., 2015; Tian et al., 2019; Zhou et al., 2017).

Leaves of cereal crops generally comprise three main parts: the blade, the sheath, and the ligular region, which serves as the boundary between the blade and sheath. The ligular region is a wedge-shaped structure containing four components: the midrib, the ligule (a fringe of epidermally derived tissue), the lamina joint, and a pair of auricles (thickened tissues connecting the blade and the sheath) (Jang, 2017; Kong et al., 2017; Li et al., 2021; Shi et al., 2024). Auricles not only provide structural support to the blade but also play a critical regulatory role in plant growth and development, particularly in determining leaf angle. Variations in auricle morphology and development affect leaf angle size, a phenomenon especially noticeable in cereal crops like corn, rice, and sorghum.

The LIGULESS1 (*LG1*) gene encodes an SQUAMOSA PROMOTER BINDING-LIKE (SPL) transcription factor. In the maize *liguless1* (*lg1*) mutant, the loss of LG1 function results in the absence of ligules and auricles, leading to a reduced leaf angle phenotype (Li et al., 2020; Moreno et al., 1997). Similarly, the maize *liguless2* (*lg2*) mutant lacks ligules and auricles, exhibits impaired lamina joint development, and shows a reduced leaf angle (Fornalé et al., 2010; Kong et al., 2017; Walsh et al., 1998; Wang et al., 2024; Xu et al., 2021). In the *increased leaf angle 1* (*Osila1*) mutant, abnormalities in vascular bundle formation during secondary cell wall biosynthesis led to an increased leaf angle (Ning et al., 2011; Sun et al., 2020).

Lignin accumulation in sclerenchyma cells provides robust support necessary for maintaining leaf erectness (Huang et al., 2021; Sun et al., 2015; Wang et al., 2020). In wheat liguleless mutants, *T. aestivum flag leaf angle 1* (*Tafla1b*) and *T. aestivum squamosa promoter binding-like 8* (*Taspl8a*), the absence of genes regulating ligule development results in reduced leaf angles (Sasaki et al., 1996; Wang et al., 2024). Research indicates that leaf angle is primarily determined by the balance between the pushing force of adaxial parenchymal cells and the mechanical support from vascular bundles and sclerenchyma cells. Furthermore, the number and size of sclerenchyma cells adjacent to vascular bundles also affect leaf angle (Dong et al., 2022; Wang et al., 2020; Zhou et al., 2017).

The phenylpropanoid biosynthesis pathway is a crucial secondary metabolic process in plants, beginning with phenylalanine as its primary substrate. This pathway synthesizes various important secondary metabolites through the catalytic actions of several enzymes, including phenylalanine ammonia-lyase (PAL). These metabolites comprise lignin, flavonoids, and coumarins (Elkind et al., 1990; Moura et al., 2010). Recent studies have increasingly shown a strong association between the phenylpropanoid biosynthesis pathway and leaf angle (Liu et al., 2024a; Mantilla-Perez and Salas Fernandez, 2017; Sakamoto et al., 2006; Yamamuro et al., 2000; Zhang et al., 2015).

The transcription factor FOUR LIPS (*OsFLP*), an *R2R3-MYB*, facilitates lignin deposition in sclerenchyma cells by promoting the expression of the phenylpropanoid biosynthesis genes *OsPAL4* and *OsPAL6* in rice (Liu et al., 2024a). In the *Osflp-1* mutant, a reduction in lignin content enhances the mechanical strength of lamina joint cells, restricts cell elongation, and significantly increases the leaf angle (Ambavaram et al., 2011; Liu et al., 2024a; Zhao et al., 2020).

Research reveals that compact varieties exhibit a significant bimodal distribution of lignin on the adaxial and abaxial sides of the lamina joint, whereas flat plant-type varieties display lignin deposition only on the abaxial side. This finding indicates that the spatial distribution pattern of lignin directly influences leaf angle (Cao et al., 2022; Miyamoto et al., 2020). However, the mechanism by which phenylpropanoid biosynthesis affects leaf angle formation in sorghum remains unclear.

This study investigates the effects of key differentially accumulated metabolites (DAMs) and differentially expressed genes (DEGs) involved in phenylpropanoid biosynthesis on leaf angle formation. We employ phenotypic analysis, cytological observation, and omics analyses comparing *el1* mutants and WT sorghum. The research aims to provide valuable insights for improving sorghum plant architecture and increasing yield, offering more precise genetic regulation strategies for breeding.

## 2 Materials and methods

### 2.1 Plant materials

The *el1* mutant originated from the sorghum inbred line BTX623, developed through mutagenesis using 0.1% ethyl methane sulfonate (EMS). The mutagenized seeds were planted at the Scientific Research Experimental Base of the Liaoning Academy of Agricultural Sciences (42°11′51″N, 123°25′9″E, 55m). The M1 generation was retained as a single plant, and *el1* with erect leaves was identified in the M2 population. The *el1* gene was stably inherited with the erect-leaf trait after 3 years of continuous self-crossing.

### 2.2 Methods

#### 2.2.1 Phenotypic analysis and cytological observation of auricles in *el1* and WT

Healthy and intact auricles from the second leaf were selected and placed in the formaldehyde-acetic acid-ethanol (FAA) fixative solution at the S1 stage. The samples were vacuum-infiltrated for 2h and subsequently stored at 4 °C for 24h. Following two rinses with 50% ethanol, the tissues were subjected to gradient dehydration using 70%, 80%, 90%, and 100% ethanol solutions, each for 30min, with three repetitions in the 100% ethanol step. Then the samples were dried using a critical point dryer (Quorum, UK), sputter-coated with gold, and observed under a Hitachi SEM 1000II scanning electron microscope for imaging.

#### 2.2.2 Metabolite extraction and metabolite analyses

In July 2023, auricle tissues from *el1* and WT were collected at the S6 stage for transcriptomic and metabolomic analyses. For each sample, auricles from the top 3rd leaves of 50 plants were pooled, with three biological replicates prepared. The samples were first lyophilized using a vacuum freeze-dryer (Scientz-100F) and subsequently ground into powder using a mixer mill set to 40Hz for 50 s. The analysis was conducted using a UPLC-ESI-MS/MS system, specifically a SHIMADZU Nexera X2 UPLC system (www.shimadzu.com.cn/) coupled with an Applied Biosystems 4500 Q TRAP mass spectrometer (www.appliedbiosystems.com.cn/).

Perform standard processing on metabolite data. Principal component analysis (PCA) and clustering heatmap of metabolites from 6 samples using R version 3.5.1DAMs were identified with thresholds of p < 0.05 and a fold change > 2. The Kyoto Encyclopedia of Genes and Genomes (KEGG) database was employed for the functional annotation of DAMs.

#### 2.2.3 RNA extraction and transcriptome sequencing

At the S6 stage, total RNA was extracted in WT and *el1*, using the RNA Easy Fast Plant Tissue RNA Rapid Extraction Kit. The integrity of the RNA was assessed with a Bioanalyzer 2100 (Agilent, CA, USA). mRNA containing polyadenylic acid (PolyA) was specifically captured with oligo(dT) magnetic beads (Dynabeads Oligo(dT), Thermo Fisher, USA). This fragmented RNA was then synthesized into cDNA using Invitrogen SuperScript™ II Reverse Transcriptase (CA, USA). Double-strand synthesis followed, employing *E. coli* DNA polymerase I and RNase H (both from NEB, USA) to convert single-stranded DNA-RNA hybrids into double-stranded DNA, while dUTP Solution (Thermo Fisher, CA, USA) was incorporated to create blunt ends on the DNA fragments. An adenine (A) base was added to each end of the double-stranded DNA to facilitate ligation with T-tailed adapters. Fragment size selection and purification were performed using magnetic beads, and the second strand was digested with the Uracil-DNA Glycosylase enzyme (NEB, MA, USA).The resulting libraries measured approximately 300 ± 50 base pairs. Paired-end sequencing (PE150) was conducted on an Illumina Novaseq™ 6000 (LC Bio Technology CO, Ltd., Hangzhou, China) following standard protocols. These clean reads were aligned to the reference genome sequence from Phytozome using the HISAT2 short-read alignment tool. Based on alignment data and gene positional information on the reference genome, the number of reads per gene was quantified. Reads per kilobase per million mapped reads (FPKM) normalized the number of mapped reads and transcript length, serving as an indicator of transcript or gene expression levels, as noted by Liu et al. (2021b). Pearson’s correlation coefficient (r), as referenced by Gang et al. (2023), was calculated to assess the significance between WT and *el1*. DEGs were identified using thresholds of p < 0.05 and |log_2_FC| ≥ 1 and subsequently analyzed through KEGG enrichment analysis.

#### 2.2.4 Analysis of phenylpropanoid biosynthesis genes by qRT-PCR

Quantitative real-time PCR (qRT-PCR) was conducted with SYBR Premix Ex Taq™ II (Tiangen Biotech Co., Ltd., Beijing, China), employing *actin* genes as the internal reference gene in sorghum. Six DEGs involved in phenylpropanoid biosynthesis were selected for validation through qPCR (**Supplementary Table S1**).

The qRT-PCR reaction system included the following components: 5 μL of SYBR Premix Ex Taq™ II, 0.5 μL of cDNA, 0.25 μL of forward primer, 0.25 μL of reverse primer, and nuclease-free water to achieve a total volume of 10 μL.

The qRT-PCR program involved the following steps: an initial pre-denaturation at 95 °C for 1min; 50 cycles consisting of denaturation at 95 °C for 5 s, annealing at 58 °C for 25 s, and extension at 72 °C for 18 s; and a final extension at 72 °C for 10min. We conducted PCR amplification using a LightCycler 480 II^®^ instrument, executing three technical replicates per sample. Analysis of the data employed the 2^-ΔΔCt^ method.

#### 2.2.5 Data statistics and analysis

The MetWare Cloud platform (https://cloud.metware.cn/) was used to conduct KEGG pathway enrichment analysis for DAMs and DEGs. We performed statistical analysis and visualization of the samples, each with three biological replicates, using GraphPad Prism 10.2 software.

## 3 Results

### 3.1 Analysis of agronomic traits and scanning electron microscopy of *el1* and WT

**Figure 1:**
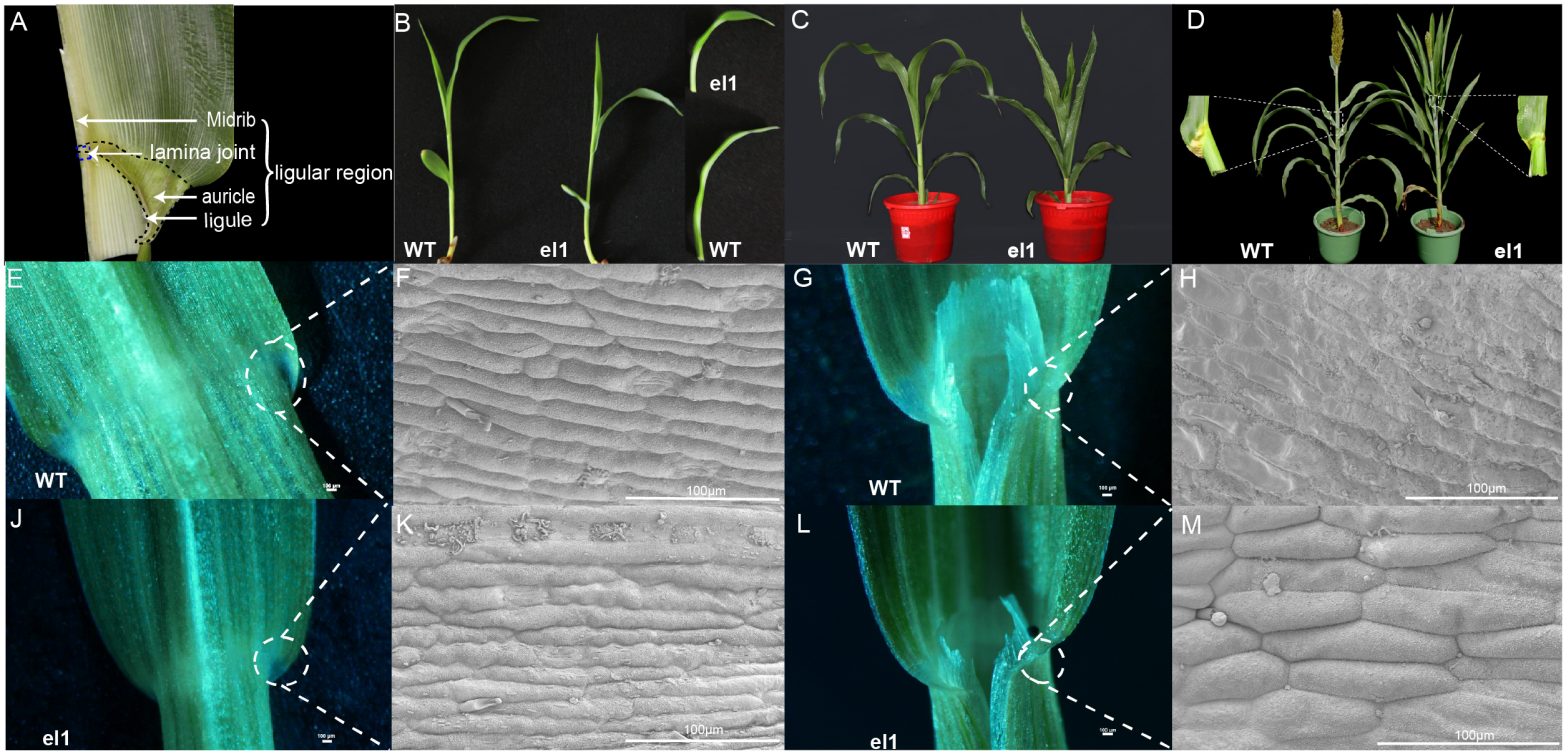
Phenotypic and cytological analysis of WT and *el1.***(A)** Leaf auricle region of the WT; **(B)**The WT and the *el1* phenotypes in the S1; **(C)** The WT and elphenotype in the S3; **(D)** The WT and the el phenotypes in the S6; **(E–M)** SEM analysis of the second leaf in the S1 for the WT and the *el1*. S1 (Stage1) - Three Leaf; S2- Five Leaf; S3- Growing Point Differentiation; S4- Flag Leaf Visible; S5- Boot; S6: Half Bloom.

In the ligule region of the WT, the auricles, ligule, and lamina joint were clearly visible (**Figure 1A**). To investigate the molecular mechanisms underlying leaf angle formation in sorghum, we chemically mutagenized the sorghum inbred line BTx623 with 0.1% EMS and identified a mutant, *el1*, with a reduced leaf angle. Compared to the WT, the *el1* exhibited shortened second leaves and erect third leaves at the S1 stage, although there was no significant difference in plant height at this stage (**Figure 1B**). The leaves of *el1* began to shrink, the upper leaves curled slightly inward, and the leaf angles were significantly reduced at the S3 stage (**Figure 1C**). At S6 stage, the auricle area had reached its maximum size. At this point, when the size of the auricles ceased to change, the leaf angle also remained stable (**Figure 1D**; **Supplementary Table S2**).

We conducted cytological observations of the second leaf in WT and *el1* during the S1 stage, focusing on the adaxial and abaxial sides of the lamina joint. Compared to WT, there was a reduction in the number of leaf auricle cells in *el1*, while their size significantly increased (**Figures 1E–M**).

### 3.2 Metabolomics identifies key metabolites in auricles involved in regulating leaf angle formation in sorghum

#### 3.2.1 Metabolome sequencing and metabolite analysis

**Figure 2:**
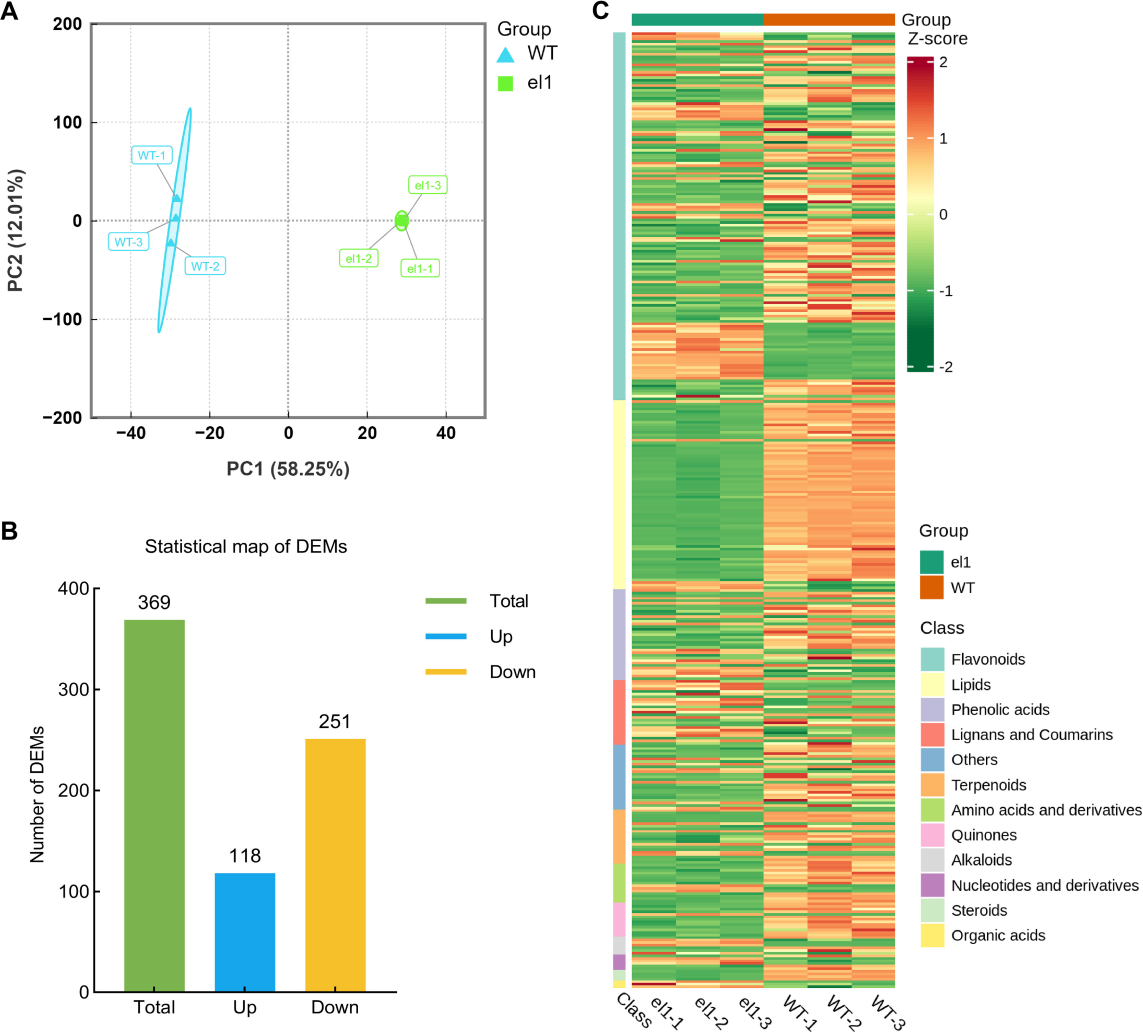
Metabolome analysis of the WT and the *el1*; **(A)** principal component analysis; **(B)** DAMs statistics map; **(C)** cluster heat map.

To clarify the impact of key DAMs auricles on leaf angle formation during development in sorghum, we employed gas chromatography-mass spectrometry (GC-MS) to detect metabolic changes in the auricles of WT and the *el1* at the S6 stage. We detected a total of 1,713 DAMs (**Supplementary Figure S1**). PCA revealed that Principal Component 1 (PC1) accounts for 58.25% of the total variance, while Principal Component 2 (PC2) accounts for 12.01% (**Figure 2A**). The reproducibility of intra-group samples was high, and samples from the different groups were well differentiated. Using thresholds of |fold change| > 2 and VIP > 1 (based on three biological replicates), we identified a total of 369 DAMs. In comparison to the WT, 118 DAMs were significantly upregulated (ratio ≥ 2, p < 0.01), and 251 DAMs were significantly downregulated (ratio ≥ 2, p < 0.01) in the *el1* (**Figure 2B**). Cluster analysis of all DAMs indicated they were predominantly enriched in flavonoids, lipids, phenolic acids, and lignans (**Figure 2C**).

#### 3.2.2 KEGG enrichment analysis of DAMs

**Figure 3:**
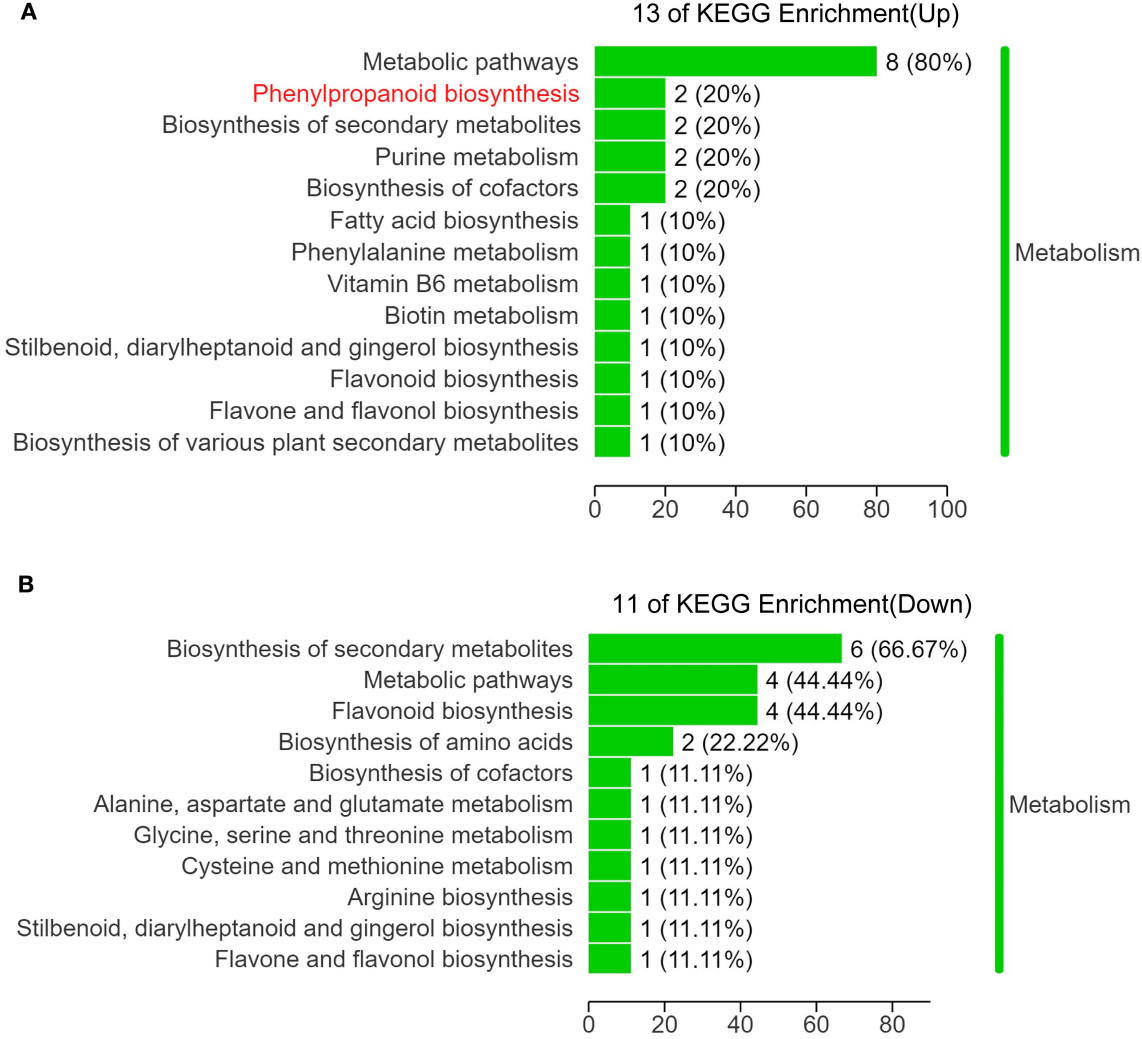
KEGG analysis of DAMs. KEGG pathway enrichment analysis for DAMs that were upregulated **(A)** and downregulated **(B)** in the *el1* compared with the WT.

To further investigate the role of DAMs in influencing leaf angle in *el1*, we conducted an enrichment analysis on DAMs present in the auricles of WT and *el1*. Among the 118 upregulated DAMs in the *el1*, 10 were functionally annotated. Whereas, among the 251 downregulated DAMs, 9 DAMs were functionally annotated, and all annotated DAMs were enriched in 18 KEGG pathways (**Supplementary Table S3**). The upregulated DAMs were mainly enriched in metabolic pathways, phenylpropanoid biosynthesis, and the biosynthesis of secondary metabolites. Specifically, eight DAMs (80%) were linked to metabolic pathways, and two DAMs (20%) each were linked to phenylpropanoid biosynthesis, secondary metabolite biosynthesis, cofactor biosynthesis, and purine metabolism. Each of the other pathways contained one DAM (10%) (**Figure 3A**). The downregulated DAMs were predominantly enriched in secondary metabolite biosynthesis, general metabolic pathways, and flavonoid biosynthesis. Specifically, six DAMs (66.67%) were enriched in secondary metabolite biosynthesis, four DAMs (44.44%) each in metabolic pathways and flavonoid biosynthesis, two DAMs (22.22%) in amino acid biosynthesis, and one DAM (11.11%) in each of the other pathways (**Figure 3B**).

### 3.3 Transcriptome sequencing identifies key regulatory genes in auricles involved in regulating leaf angle formation in sorghum

#### 3.3.1 Transcriptome sequencing and gene expression analysis

**Figure 4:**
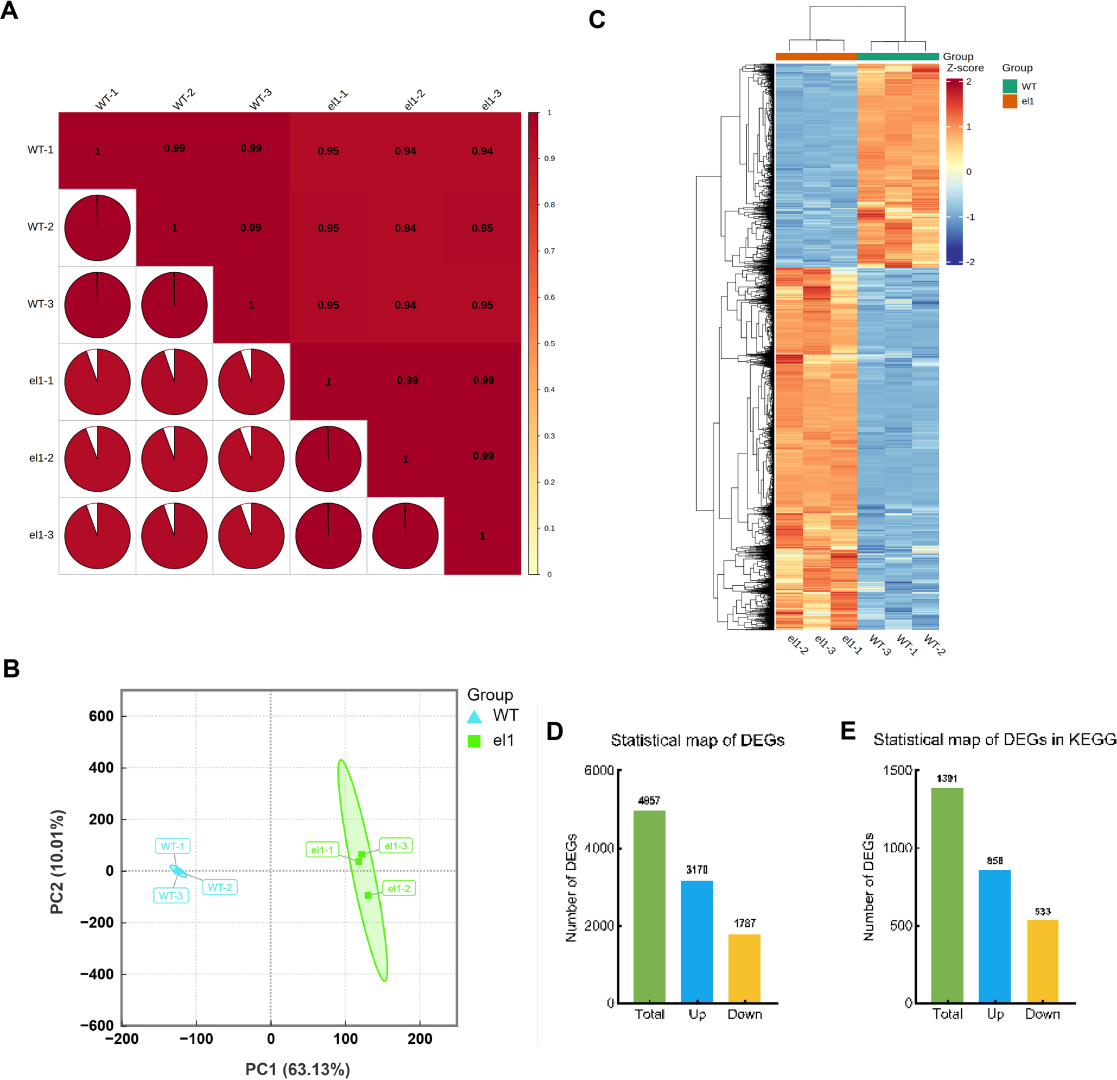
Transcriptome analysis of the WT and the *el1*. **(A)** Correlation heat map; **(B)** Principal component analysis; **(C)** Cluster heat map; **(D)** DEGs statistical map. **(E)** DEGs statistical map in KEGG.

To clarify the impact of DEGs on leaf angle in the auricles of sorghum, we conducted RNA-seq analysis on the auricles of the WT and *el1* at the S6 stage. Details of transcriptome sequencing data assembly and analysis for the WT and *el1* are provided in **Table 1**. A total of six libraries were created for each group of samples. Following the removal of low-quality data, we obtained 55.47 Gb of clean data, with each sample contributing at least 8.30 Gb. The Q20 scores ranged from 98.04% to 98.18%, Q30 scores from 94.37% to 94.74%, and GC content ranged from 54.83% to 57.26%. Clean reads from each sample were aligned with the sorghum reference genome sequence (https://phytozome-next.jgi.doe.gov/info/Sbicolor_v3_1_1), achieving an alignment efficiency exceeding 82.11%. More than 80.16% of clean reads in each sample uniquely mapped to the reference genome (**Table 1**). We used Pearson’s correlation coefficients to evaluate biological relevance, with pairwise comparisons between the groups yielding R² values above 0.99, indicating high consistency among biological replicates (**Figure 4A**). PCA showed that PC1 accounted for 63.13% of the total variance and PC2 for 10.01%. Samples within groups clustered together, while samples between groups were clearly distinguishable, demonstrating good consistency within groups and significant differences between the two varieties (**Figure 4B**). These results confirm that the RNA-seq data obtained are of high quality and suitable for subsequent analysis.

**Table 1:** Statistics and comparative analysis of quality control data.

Sample	Raw reads	Clean reads	Clean base (G)	Read mapped (%)	Unique mapped (%)	Q20 (%)	Q30 (%)	GC content (%)
WT-1	64157110	61527916	9.23	82.46	80.51	98.18	94.69	54.83
WT-2	63093128	60643092	9.10	82.11	80.16	98.04	94.37	54.84
WT-3	70517422	67820872	10.17	82.51	80.55	98.18	94.74	54.85
el-1	67913814	65122214	9.77	89.41	87.42	98.07	94.53	57.14
el-2	58011080	55365154	8.30	89.12	87.16	98.12	94.57	57.24
el-3	61564762	59342136	8.90	89.37	87.42	98.07	94.52	57.26

A total of 201,436 genes were detected in the WT and the *el1* (**Supplementary Figure S2**). To identify genes potentially involved in the formation of leaf angles in sorghum, we screened for DEGs using criteria of a fold change greater than 2 and a false discovery rate (FDR) less than 0.05, based on three biological replicates. We conducted cluster heat map analysis on these DEGs, identifying a total of 4,957 DEGs, which were organized into ten groups (**Figure 4C**). Of these, 3,170 DEGs were upregulated, while 1,787 were downregulated (**Figure 4D**). Among them, 858 upregulated and 533 downregulated DEGs were functionally annotated (**Figure 4E**).

#### 3.3.2 KEGG enrichment analysis of DEGs

**Figure 5:**
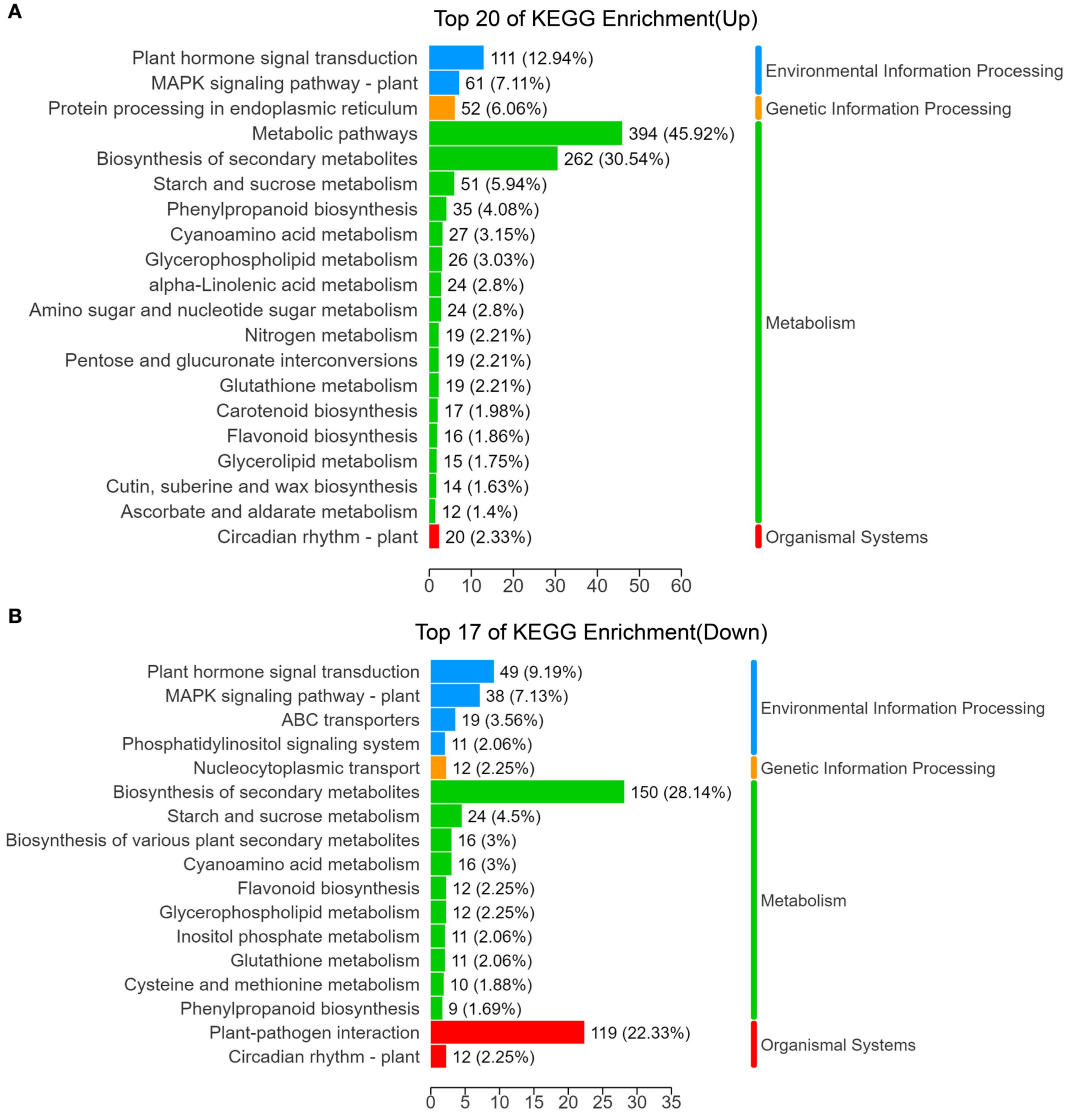
KEGG analysis of DEGs. KEGG pathway enrichment analysis for DEGs that were upregulated **(A)** or downregulated **(B)** in the *el1* compared with the WT.

To further investigate the roles of DEGs in leaf angle formation in sorghum, we performed KEGG enrichment analysis on the annotated DEGs (**Figure 5A**). The analysis revealed that the upregulated DEGs were primarily enriched in five pathways: metabolic pathways, biosynthesis of secondary metabolites, plant hormone signal transduction, starch and sucrose metabolism, and phenylpropanoid biosynthesis. Specifically, 394 DEGs (45.92%) were associated with metabolic pathways, 262 DEGs (30.54%) with biosynthesis of secondary metabolites, 111 DEGs (12.94%) with plant hormone signal transduction, and 35 DEGs (4.08%) with phenylpropanoid biosynthesis (**Figure 5A**).

Conversely, the downregulated DEGs were mainly enriched in pathways such as biosynthesis of secondary metabolites, plant-pathogen interaction, plant hormone signal transduction, and phenylpropanoid metabolism. In particular, 150 DEGs (28.14%) were involved in the biosynthesis of secondary metabolites, 119 DEGs (22.33%) in plant-pathogen interaction, 49 DEGs (9.19%) in plant hormone signal transduction, and 9 DEGs (1.69%) in phenylpropanoid metabolism (**Figure 5B**; **Supplementary Figure S3**).

### 3.4 Correlation analysis between DAMs and DEGs in the phenylpropanoid biosynthesis during the formation of leaf angles in sorghum

**Figure 6:**
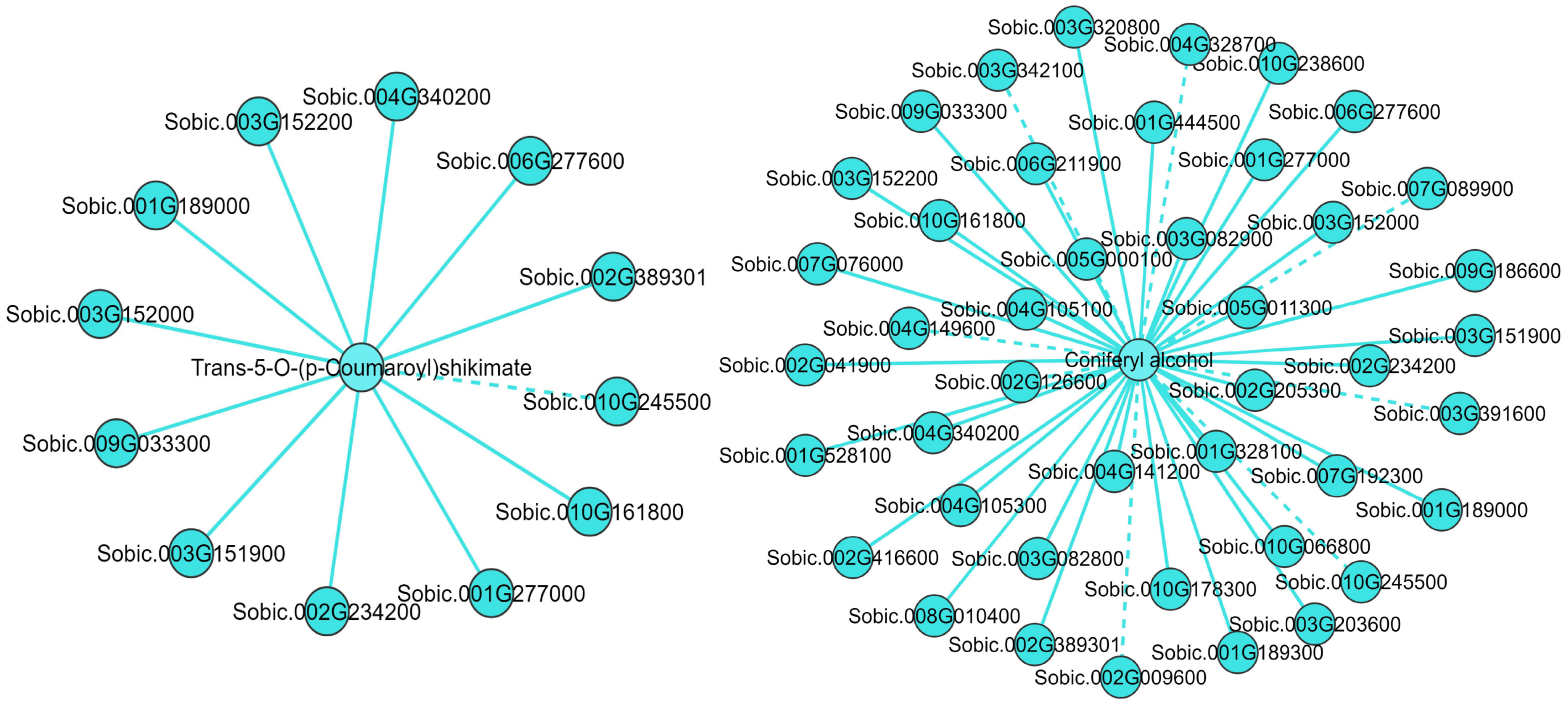
Correlation network of DAMs and DEGs in the phenylpropanoid biosynthesis during the formation of sorghum leaf angles. Solid lines represent positive correlations, and dashed lines represent negative correlations.

Pearson’s correlation analysis was used to assess the correlation between DEGs and DAMs in phenylpropanoid biosynthesis. The analysis revealed that 43 DEGs were associated with coniferyl alcohol. Of these, 35 DEGs showed a positive correlation, while 8 DEGs exhibited a negative correlation. Additionally, 12 DEGs were linked to trans-5-O-(p-coumaroyl) shikimate; among these, 11 DEGs were positively correlated, and 1 DEG was negatively correlated. Importantly, all 12 DEGs connected with trans-5-O-(p-coumaroyl) shikimate regulation also played a role in the regulation of coniferyl alcohol (**Figure 6**; **Supplementary Table S4**). These findings suggest that the DAMs and DEGs may significantly contribute to the formation of the leaf angle in sorghum.

### 3.5 Analysis of the phenylpropanoid biosynthesis during the formation of leaf angles in sorghum

**Figure 7:**
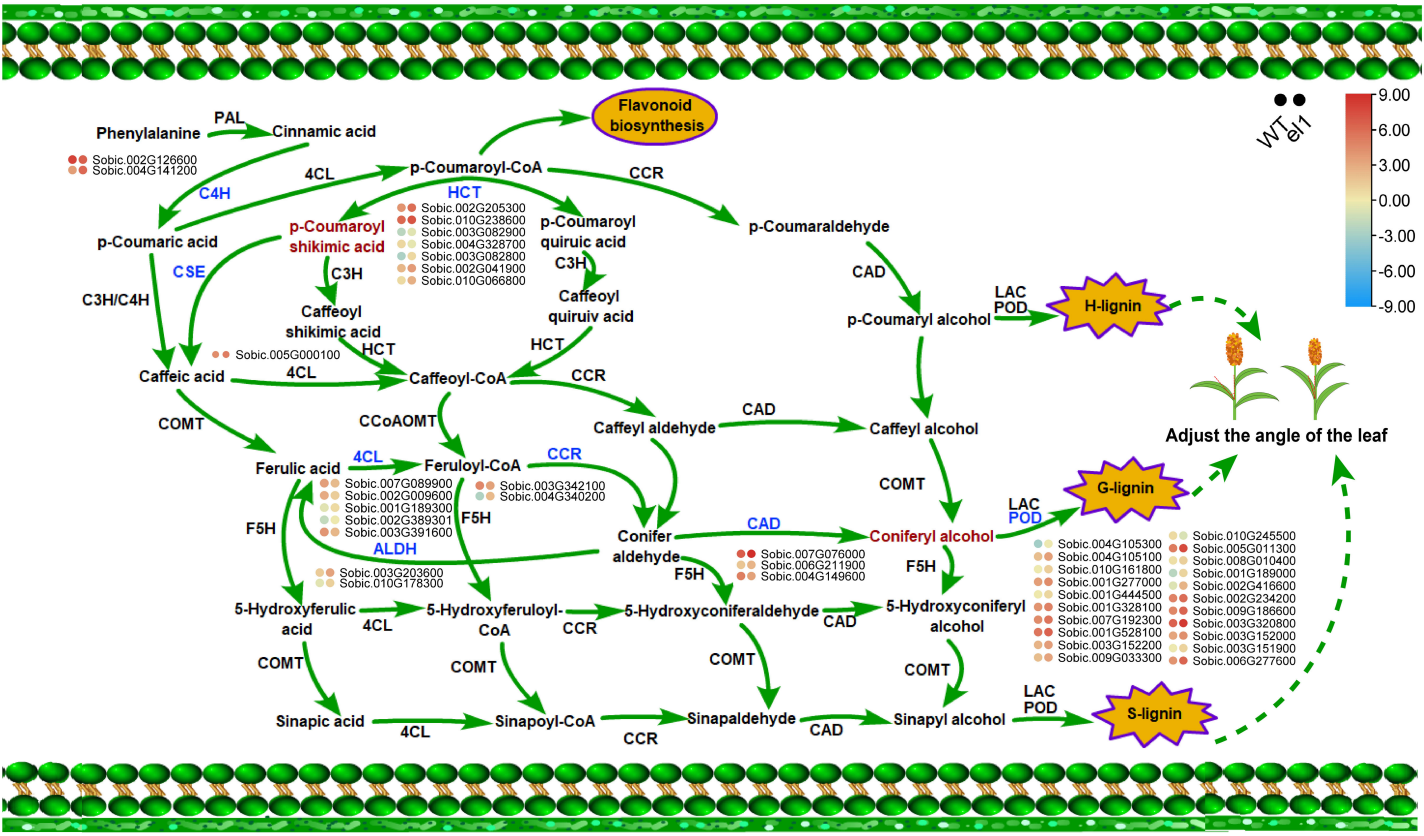
The phenylpropanoid biosynthesis during the formation of the leaves angle in sorghum.

In conjunction with DEGs identification and correlation analysis, genes associated with phenylpropanoid biosynthesis were found to be differentially expressed in auricles of sorghum (**Figure 7**). The phenylpropanoid biosynthesis pathway begins with phenylalanine, which is catalyzed by PAL and cinnamate 4-hydroxylase (C4H) to form p-coumaric acid. Two DEGs were identified at this stage. Compared to the WT, the expression levels of *Sobic.002G126699* and *Sobic.004G141200* were both downregulated. In the presence of 4CL catalysis, p-Coumaroyl-CoA is generated, and hydroxycinnamoyl-CoA shikimate/quinate transferase (HCT) serves as the key enzyme directing lignin metabolism. HCT converts p-coumaroyl-CoA into p-coumaryol shikimic acid. Seven DEGs were detected in this process: *Sobic.002G205300*, *Sobic.010G238600*, *Sobic.013G082900*, *Sobic.004G328700*, *Sobic.003G082800*, *Sobic.002G041900*, and *Sobic.010G066800*. Compared to the WT, all these DEGs were upregulated except *Sobic.004G328700*.

P-Coumaryol shikimic acid is subsequently converted to ferulic acid through the actions of caffeic acid O-methyltransferase (CSE) and caffeoyl-CoA O-methyltransferase (COMT). Ferulic acid is further transformed into feruloyl-CoA by 4CL. Five DEGs were detected during this stage: *Sobic.007G089900*, *Sobic.002G009600*, *Sobic.001G189300*, *Sobic.002G389301*, and *Sobic.003G3091600*. Notably, the expression of *Sobic.007G089900* and *Sobic.002G009600* decreased significantly. Cinnamoyl-CoA reductase (CCR) catalyzes feruloyl-CoA to synthesize conifer aldehyde directly, with two genes (*Sobic.004G342100* and *Sobic.004G340200*) identified in this process. Cinnamyl alcohol dehydrogenase (CAD) further catalyzes conifer aldehyde to form coniferyl alcohol, with three DEGs (*Sobic.007G076000*, *Sobic.006G211900*, and *Sobic.004G149600*) identified. In the *el1*, *Sobic.004G149600* was downregulated.

Coniferyl alcohol is converted into G-lignin monomers through the action of peroxidase (POD). Among the 21 DEGs directly related to POD, only *Sobic.010G245500* showed a downregulated trend, with the expression of all other genes being upregulated. These findings suggest that significant changes in the expression of these genes can affect lignin synthesis, thereby influencing the formation of sorghum leaf angle.

### 3.6 qRT-PCR analysis of genes in the phenylpropanoid biosynthesis

**Figure 8:**
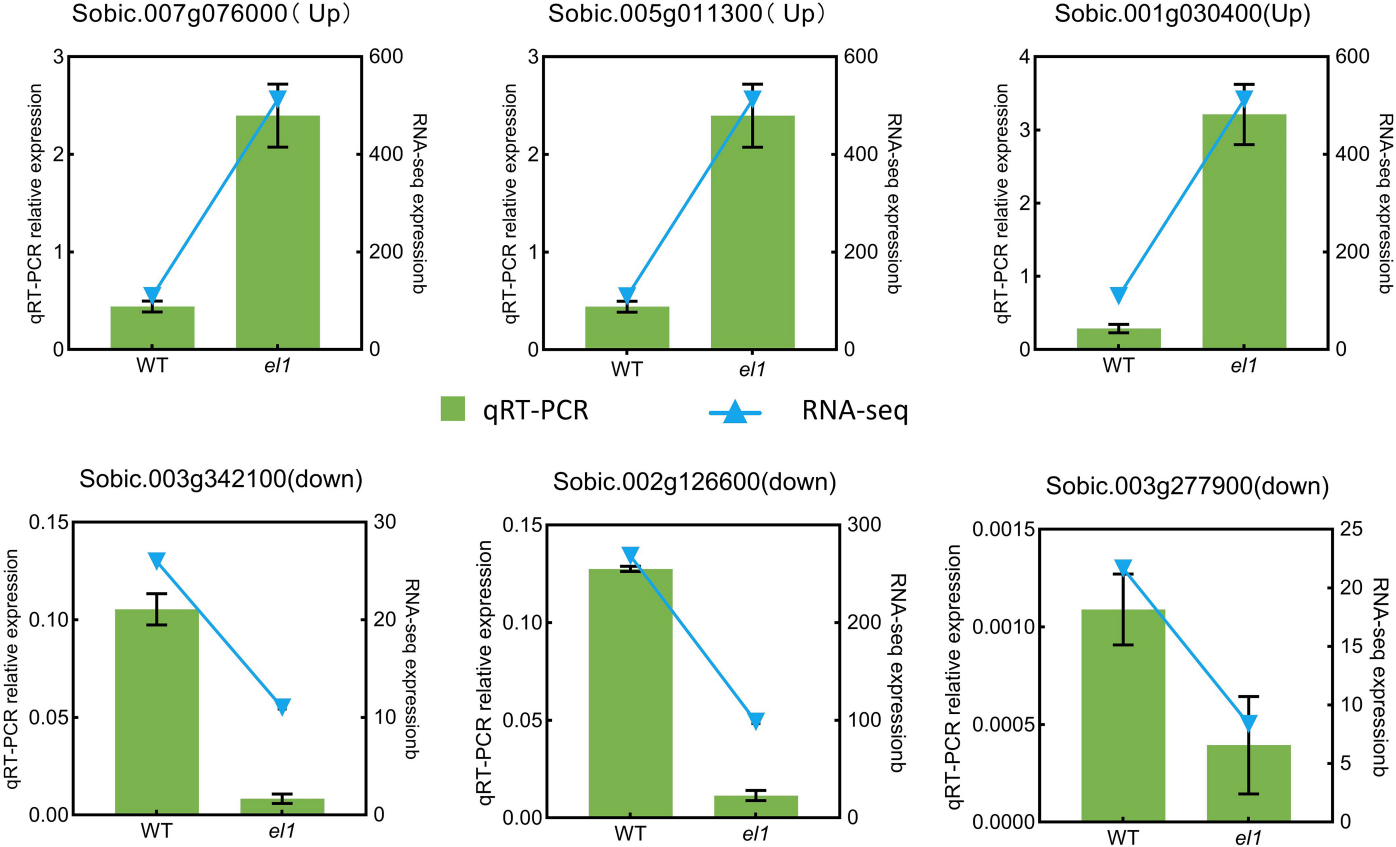
qRT-PCR expression analysis of DEGs.

To verify the accuracy of DEGs identified by RNA-seq in the phenylpropanoid biosynthesis pathway, we conducted a qRT-PCR analysis on six randomly selected DEGs involved in phenylalanine biosynthesis. The qRT-PCR results demonstrated expression trends consistent with the transcriptome data analysis (**Figure 8**), indicating the reliability of the transcriptomic findings.

## 4 Discussion

Leaf angle, a crucial trait of ideal plant architecture in crops, directly influences crop yield (Mantilla-Perez and Salas Fernandez, 2017; Yang et al., 2023). Most current studies on crop leaf angles focus on hormone regulation and the associated signaling pathways (Chen et al., 2018; Huang et al., 2023; Liu et al., 2021b; Luo et al., 2016; Sakamoto et al., 2006; Yamamuro et al., 2000). This study elucidates the mechanisms influencing leaf angle formation in sorghum by integrating cytology, transcriptomics, and metabolomic analyses. The auricle, which connects the leaf blade to sheath, plays a crucial role in determining leaf angle. In maize, ligule mutants lacking ligules and auricles exhibit reduced leaf angles. The maize *brd1-m1* mutant exhibits enlarged auricles and indistinct boundaries between the leaf blade and sheath (Tanabe et al., 2005), leading to increased leaf angles. In our study, an EMS induced mutant with reduced leaf angles, named *el1*, was obtained. Cytological analysis of the second leaf at the S1 stage revealed that, compared with the WT, *el1* had significantly fewer and smaller auricle cells on the adaxial and abaxial sides (**Figures 1E–M**). By the S6 stage, the auricle size no longer changed, and those in the *el1* were significantly smaller than in the WT (**Figure 1D**).

We conducted KEGG enrichment analysis on upregulated and downregulated DAMs in the auricle metabolome of WT and *el1* at the S6 stage in sorghum (**Figure 5A**). The analysis revealed that upregulated DAMs were enriched in 13 metabolic pathways. Notably, two DAMs were concentrated in the phenylpropanoid biosynthesis: trans-5-O-(p-Coumaroyl)shikimate and coniferyl alcohol. In contrast, downregulated DAMs were primarily enriched in 11 metabolic pathways. These included the biosynthesis of secondary metabolites, general metabolic pathways, and flavonoid biosynthesis.

Transcriptome analysis identified a total of 1,391 DEGs with functional annotations, comprising 858 upregulated and 533 downregulated DEGs. These DEGs were primarily enriched in pathways including metabolic pathways, secondary metabolite biosynthesis, plant hormone signal transduction, and phenylpropanoid biosynthesis (**Figure 5A**). The KEGG enrichment results underscore the significance of phenylpropanoid biosynthesis in the formation of leaf angle in sorghum.

Trans-5-O-(p-Coumaroyl)shikimate is a crucial intermediate in phenylpropanoid biosynthesis. It is a lipid compound synthesized through the catalytic action of HCT, which links p-coumaroyl-CoA with shikimic acid. This reaction channels phenylpropanoid metabolism toward the lignin synthesis pathway. Subsequently, C3H (caffeoyl-CoA 3’-hydroxylase) introduces a hydroxyl group at the C3 position of trans-5-O-(p-coumaroyl)shikimate, converting it into 5-O-caffeoyl shikimate. This compound is further metabolized into caffeoyl-CoA. Caffeoyl-CoA is then methylated by CCoAOMT (caffeoyl-CoA O-methyltransferase) to form feruloyl-CoA, which is ultimately reduced to coniferyl alcohol by CCR and CAD (Chen et al., 2024; Elkind et al., 1990; Su et al., 2020; Zakzeski et al., 2010; Zhong et al., 2000). Coniferyl alcohol is a critical monomeric precursor for lignin synthesis, playing a direct role in the mechanical reinforcement of plant cell walls and the regulation of stress resistance (Liu et al., 2018; Van Acker et al., 2013). In rice, *hct* leads to reduced lignin synthesis, decreased sheath support, and drooping leaves with increased angles (Hou et al., 2019). Conversely, overexpression of the *CCR* gene results in increased lignin deposition and upright leaves (Tanaka et al., 2009; Tobimatsu and Schuetz, 2019).

Integrated metabolomics and transcriptomic analyses identified 12 DEGs involving in regulating the expression of trans-5-O-(p-Coumaroyl)shikimate, all of which are also associated with the regulation of coniferyl alcohol. This finding further validates that the metabolic level of trans-5-O-(p-Coumaroyl)shikimate is crucial for the production efficiency of coniferyl alcohol.

Plant hormones can alter leaf angle by influencing the mechanical support force at the lamina joints (Liu et al., 2024b). In rice, *OsFLP* directly regulates the transcription of the *OsPAL*-family genes, and modulates rice leaf angle through affecting lignin deposition. The transcription of *OsFLP* is controlled by Oryza sativa BRASSINAZOLE RESISTANT 1 (*OsBZR1*), a signaling factor in the brassinosteroid (BR) pathway. Meanwhile, *OsFLP* inhibits the transcription of GLYCOGEN SYNTHASE KINASE-3 (OsGSK3), which in turn affects the phosphorylation status of OsBZR1. Collectively, these processes form a mechanism in BR signal transduction that regulates lignin biosynthesis to alter rice leaf angle (Liu et al., 2024a). Similarly, AUXIN RESPONSE FACTOR 6 (OsARF6) and AUXIN RESPONSE FACTOR 17 (OsARF17) regulate the flag leaf angle in rice by controlling the biosynthesis of secondary cell walls at the rice lamina joints (Huang et al., 2021).

Consequently, we infer that during leaf angle formation in sorghum, the size and morphology of the leaf angle may be influenced by the regulation of key metabolite production and the expression of essential DEGs in the phenylpropanoid biosynthesis pathway. This discovery not only provides valuable insights into the molecular mechanisms underlying leaf angle formation but also offers a theoretical foundation for future improvements in sorghum plant architecture.

## Data Availability

The datapresented in the study are deposited in the NCBI repository, accession number PRJNA1347625.

## References

[B1] Ambavaram M. M. Krishnan A. Trijatmiko K. R. Pereira A. (2011). Coordinated activation of cellulose and repression of lignin biosynthesis pathways in rice. Plant Physiol. 155, 916–931. doi: 10.1104/pp.110.168641, PMID: 21205614 PMC3032476

[B2] Cao Y. Zhong Z. Wang H. Shen R. (2022). Leaf angle: a target of genetic improvement in cereal crops tailored for high-density planting. Plant Biotechnol. J. 20, 426–436. doi: 10.1111/pbi.13780, PMID: 35075761 PMC8882799

[B3] Chen H. Gong X. Guo Y. Yu J. Li W.-X. Du Q. (2024). ZmbZIP27 regulates nitrogen-mediated leaf angle by modulating lignin deposition in maize. Crop J. 12, 1404–1413. doi: 10.1016/j.cj.2024.09.004

[B4] Chen S.-H. Zhou L.-J. Xu P. Xue H.-W. (2018). SPOC domain-containing protein Leaf inclination3 interacts with LIP1 to regulate rice leaf inclination through auxin signaling. PLoS Genet. 14, e1007829. doi: 10.1371/journal.pgen.1007829, PMID: 30496185 PMC6289470

[B5] Dong S. Lu X. Li J. Han J. (2022). Phenotypic identification and genetic analysis of a sorghum erect leaf mutant. J. Plant Genet. Resour. 23, 177–182. doi: 10.13430/j.cnki.jpgr.20210503001

[B6] Elkind Y. Edwards R. Mavandad M. Hedrick S. A. Ribak O. Dixon R. A. . (1990). Abnormal plant development and down-regulation of phenylpropanoid biosynthesis in transgenic tobacco containing a heterologous phenylalanine ammonia-lyase gene. Proc. Natl. Acad. Sci. U.S.A. 87, 9057–9061. doi: 10.1073/pnas.87.22.9057, PMID: 11607118 PMC55100

[B7] Fornalé S. Shi X. Chai C. Encina A. Irar S. Capellades M. . (2010). ZmMYB31 directly represses maize lignin genes and redirects the phenylpropanoid metabolic flux. Plant J. 64, 633–644. doi: 10.1111/j.1365-313x.2010.04363.x, PMID: 21070416

[B8] Gang S. Zhang S. Lu X. Gao Z. Li J. (2023). Phenotypic identification and transcriptome analysis of the narrow leaf mutant nal1 in sorghum. J. Shenyang Agric. Univ. 54, 129–139. doi: 10.3969/j.issn.1000-1700.2023.02.001

[B9] Gao Z. Gang S. Lu X. Wang P. Li Z. Wang Y. . (2024). Mapping of the sorghum yellow seed gene yellow seed 2 and analysis of candidate genes. J. Plant Genet. Resour. 25, 1565–1572. doi: 10.13430/j.cnki.jpgr.20231221002

[B10] Guo J. Li W. Shang L. Wang Y. Yan P. Bai Y. . (2021). OsbHLH98 regulates leaf angle in rice through transcriptional repression of OsBUL1. New Phytol. 230, 1953–1966. doi: 10.1111/nph.17303, PMID: 33638214

[B11] Hou Y. Wang Y. Tang L. Tong X. Wang L. Liu L. . (2019). SAPK10-mediated phosphorylation on WRKY72 releases its suppression on jasmonic acid biosynthesis and bacterial blight resistance. Iscience 16, 499–510. doi: 10.1016/j.isci.2019.06.009, PMID: 31229897 PMC6593165

[B12] Huang G. Hu H. van de Meene A. Zhang J. Dong L. Zheng S. . (2021). AUXIN RESPONSE FACTORS 6 and 17 control the flag leaf angle in rice by regulating secondary cell wall biosynthesis of lamina joints. Plant Cell 33, 3120–3133. doi: 10.1093/plcell/koab175, PMID: 34245297 PMC8462825

[B13] Huang P. Zhao J. Hong J. Zhu B. Xia S. Zhu E. . (2023). Cytokinins regulate rice lamina joint development and leaf angle. Plant Physiol. 191, 56–69. doi: 10.1093/plphys/kiac401, PMID: 36031806 PMC9806582

[B14] Jang S. (2017). A novel trimeric complex in plant cells that contributes to the lamina inclination of rice. Plant Signal. Behav. 12, e1274482. doi: 10.1080/15592324.2016.1274482, PMID: 28029278 PMC5289517

[B15] Jiao Y. Wang Y. Xue D. Wang J. Yan M. Liu G. . (2010). Regulation of OsSPL14 by OsmiR156 defines ideal plant architecture in rice. Nat. Genet. 42, 541–544. doi: 10.1038/ng.591, PMID: 20495565

[B16] Kong F. Zhang T. Liu J. Heng S. Shi Q. Zhang H. . (2017). Regulation of leaf angle by auricle development in maize. Mol. Plant 10, 516–519. doi: 10.1016/j.molp.2017.02.001, PMID: 28216423

[B17] Li J. Dong S. Li W. Zhang L. Lu X. (2021). Phenotypic identification and genetic analysis of the sorghum leaf angle mutant LA1. J. Shenyang Agric. Univ. 52, 460–466. doi: 10.3969/j.issn.1000-1700.2021.04.010

[B18] Li X. Wu P. Lu Y. Guo S. Zhong Z. Shen R. . (2020). Synergistic interaction of phytohormones in determining leaf angle in crops. Int. J. Mol. Sci. 21, 5052. doi: 10.3390/ijms21145052, PMID: 32709150 PMC7404121

[B19] Liu Q. Luo L. Zheng L. (2018). Lignins: biosynthesis and biological functions in plants. Int. J. Mol. Sci. 19, 335. doi: 10.3390/ijms19020335, PMID: 29364145 PMC5855557

[B20] Liu Z. Mei E. Tian X. He M. Tang J. Xu M. . (2021b). OsMKKK70 regulates grain size and leaf angle in rice through the OsMKK4-OsMAPK6-OsWRKY53 signaling pathway. J. Integr. Plant Biol. 63, 2043–2057. doi: 10.1111/jipb.13174, PMID: 34561955

[B21] Liu F. Song Q. Zhao J. Mao L. Bu H. Hu Y. . (2021a). Canopy occupation volume as an indicator of canopy photosynthetic capacity. New Phytol. 232, 941–956. doi: 10.1111/nph.17611, PMID: 34245568

[B22] Liu G. Z. Yang Y. S. Liu W. M. Guo X. X. Xie R. Z. Ming B. . (2022). Optimized canopy structure improves maize grain yield and resource use efficiency. Food Energy Secur. 11, e375. doi: 10.1002/fes3.375

[B23] Liu H. Zhang J. Wang J. Fan Z. Qu X. Yan M. . (2024a). The rice R2R3 MYB transcription factor FOUR LIPS connects brassinosteroid signaling to lignin deposition and leaf angle. Plant Cell 36, 4768–4785. doi: 10.1093/plcell/koae251, PMID: 39259275 PMC11530771

[B24] Liu L. Zhao L. Liu Y. Zhu Y. Chen S. Yang L. . (2024b). Transcription factor OsWRKY72 controls rice leaf angle by regulating LAZY1-mediated shoot gravitropism. Plant Physiol. 195, 1586–1600. doi: 10.1093/plphys/kiae159, PMID: 38478430

[B25] Luo X. Zheng J. Huang R. Huang Y. Wang H. Jiang L. . (2016). Phytohormones signaling and crosstalk regulating leaf angle in rice. Plant Cell Rep. 35, 2423–2433. doi: 10.1007/s00299-016-2052-5, PMID: 27623811

[B26] Mantilla-Perez M. B. Salas Fernandez M. G. (2017). Differential manipulation of leaf angle throughout the canopy: current status and prospects. J. Exp. Bot. 68, 5699–5717. doi: 10.1093/jxb/erx378, PMID: 29126242

[B27] Miyamoto T. Tobimatsu Y. Umezawa T. (2020). MYB-mediated regulation of lignin biosynthesis in grasses. Curr. Plant Biol. 24, 100174. doi: 10.1016/j.cpb.2020.100174

[B28] Moldenhauer K. A. Gibbons J. H. (2003). “ Rice morphology and development,” in Rice: Origin, History, Technology, and Production. Eds. Smith C. W. Dilday R. H. ( Wiley Series in Crop Science, Texas), 103–128.

[B29] Moreno M. A. Harper L. C. Krueger R. W. Dellaporta S. L. Freeling M. (1997). Liguleless1 encodes a nuclear-localized protein required for induction of ligules and auricles during maize leaf organogenesis. Genes Dev. 11, 616–628. doi: 10.1101/gad.11.5.616, PMID: 9119226

[B30] Moura J. C. M. S. Bonine C. A. V. De Oliveira Fernandes Viana J. Dornelas M. C. Mazzafera P. (2010). Abiotic and biotic stresses and changes in the lignin content and composition in plants. J. Integr. Plant Biol. 52, 360–376. doi: 10.1111/j.1744-7909.2010.00892.x, PMID: 20377698

[B31] Ning J. Zhang B. Wang N. Zhou Y. Xiong L. (2011). Increased leaf angle1, a Raf-like MAPKKK that interacts with a nuclear protein family, regulates mechanical tissue formation in the Lamina joint of rice. Plant Cell 23, 4334–4347. doi: 10.1105/tpc.111.093419, PMID: 22207574 PMC3269869

[B32] Sakamoto T. Morinaka Y. Ohnishi T. Sunohara H. Fujioka S. Ueguchi-Tanaka M. . (2006). Erect leaves caused by brassinosteroid deficiency increase biomass production and grain yield in rice. Nat. Biotechnol. 24, 105–109. doi: 10.1038/nbt1173, PMID: 16369540

[B33] Sasaki M. Yamamoto Y. Matsumoto H. (1996). Lignin deposition induced by aluminum in wheat (Triticum aestivum) roots. Physiol. Plant 96, 193–198. doi: 10.1111/j.1399-3054.1996.tb00201.x

[B34] Shi Q. Xia Y. Wang Q. Lv K. Yang H. Cui L. . (2024). Phytochrome B interacts with LIGULELESS1 to control plant architecture and density tolerance in maize. Mol. Plant 17, 1255–1271. doi: 10.1016/j.molp.2024.06.014, PMID: 38946140

[B35] Su N. Ling F. Xing A. Zhao H. Zhu Y. Wang Y. . (2020). Lignin synthesis mediated by CCoAOMT enzymes is required for the tolerance against excess Cu in Oryza sativa. Environ. Exp. Bot. 175, 104059. doi: 10.1016/j.envexpbot.2020.104059

[B36] Sun S. Chen D. Li X. Qiao S. Shi C. Li C. . (2015). Brassinosteroid signaling regulates leaf erectness in Oryza sativa *via* the control of a specific U-type cyclin and cell proliferation. Dev. Cell 34, 220–228. doi: 10.1016/j.devcel.2015.05.019, PMID: 26190148

[B37] Sun X. Ma Y. Yang C. Li J. (2020). Rice OVATE family protein 6 regulates leaf angle by modulating secondary cell wall biosynthesis. Plant Mol. Biol. 104, 249–261. doi: 10.1007/s11103-020-01039-2, PMID: 32715397

[B38] Tanabe S. Ashikari M. Fujioka S. Takatsuto S. Yoshida S. Yano M. . (2005). A novel cytochrome P450 is implicated in brassinosteroid biosynthesis *via* the characterization of a rice dwarf mutant, dwarf11, with reduced seed length. Plant Cell 17, 776–790. doi: 10.1105/tpc.104.024950, PMID: 15705958 PMC1069698

[B39] Tanaka A. Nakagawa H. Tomita C. Shimatani Z. Ohtake M. Nomura T. . (2009). BRASSINOSTEROID UPREGULATED1, encoding a helix-loop-helix protein, is a novel gene involved in brassinosteroid signaling and controls bending of the lamina joint in rice. Plant Physiol. 151, 669–680. doi: 10.1104/pp.109.140806, PMID: 19648232 PMC2754635

[B40] Tian J. Wang C. Xia J. Wu L. Xu G. Wu W. . (2019). Teosinte ligule allele narrows plant architecture and enhances high-density maize yields. Science 365, 658–664. doi: 10.1126/science.aax5482, PMID: 31416957

[B41] Tobimatsu Y. Schuetz M. (2019). Lignin polymerization: how do plants manage the chemistry so well? Curr. Opin. Biotech. 56, 75–81. doi: 10.1016/j.copbio.2018.10.001, PMID: 30359808

[B42] Van Acker R. Vanholme R. Storme V. Mortimer J. C. Dupree P. Boerjan W. (2013). Lignin biosynthesis perturbations affect secondary cell wall composition and saccharification yield in Arabidopsis thaliana. Biotechnol. Biofuels 6, 1–17. doi: 10.1186/1754-6834-6-46, PMID: 23622268 PMC3661393

[B43] Walsh J. Waters C. A. Freeling M. (1998). The maize gene liguleless2 encodes a basic leucine zipper protein involved in the establishment of the leaf blade-sheath boundary. Genes Dev. 12, 208–218. doi: 10.1101/gad.12.2.208, PMID: 9490265 PMC316436

[B44] Wang Q. Guo Q. Shi Q. Yang H. Liu M. Niu Y. . (2024). Histological and single-nucleus transcriptome analyses reveal the specialized functions of ligular sclerenchyma cells and key regulators of leaf angle in maize. Mol. Plant 17, 920–934. doi: 10.1016/j.molp.2024.05.001, PMID: 38720461

[B45] Wang R. Liu C. Li Q. Chen Z. Sun S. Wang X. (2020). Spatiotemporal resolved leaf angle establishment improves rice grain yield *via* controlling population density. Iscience 23, 101489. doi: 10.1016/j.isci.2020.101489, PMID: 32898833 PMC7486458

[B46] Xu J. Wang J. Xue H. Zhang G. (2021). Leaf direction: lamina joint development and environmental responses. Plant Cell Environ. 44, 2441–2454. doi: 10.1111/pce.14065, PMID: 33866581

[B47] Yamamuro C. Ihara Y. Wu X. Noguchi T. Fujioka S. Takatsuto S. . (2000). Loss of function of a rice brassinosteroid insensitive1 homolog prevents internode elongation and bending of the lamina joint. Plant Cell 12, 1591. doi: 10.2307/3871176, PMID: 11006334 PMC149072

[B48] Yang X. Li R. Jablonski A. Stovall A. Kim J. Yi K. . (2023). Leaf angle as a leaf and canopy trait: rejuvenating its role in ecology with new technology. Ecol. Lett. 26, 1005–1020. doi: 10.1111/ele.14215, PMID: 37078440

[B49] Yonemaru J. I. Ando T. Mizubayashi T. Kasuga S. Matsumoto T. Yano M. (2009). Development of genome-wide simple sequence repeat markers using whole-genome shotgun sequences of sorghum (Sorghum bicolor (L.) Moench). DNA Res. 16, 187–193. doi: 10.1093/dnares/dsp005, PMID: 19363056 PMC2695772

[B50] Zakzeski J. Bruijnincx P. C. A. Jongerius A. L. Weckhuysen B. M. (2010). The catalytic valorization of lignin for the production of renewable chemicals. Chem. Rev. 110, 3552–3599. doi: 10.1021/cr900354u, PMID: 20218547

[B51] Zhang C. Ding G. Ni X. Liu T. Chen G. Zhao G. (2013). Identification of resistance to sorghum head smut in brewing sorghum varieties, combinations, and parents. J. Plant Prot. 40, 219–224. doi: 10.13802/j.cnki.zwbhxb.2013.03.017

[B52] Zhang S. Wang S. Xu Y. Yu C. Shen C. Qian Q. . (2015). The auxin response factor, OsARF 19, controls rice leaf angles through positively regulating OsGH 3–5 and OsBRI 1. Plant Cell Environ. 38, 638–654. doi: 10.1111/pce.12397, PMID: 24995795

[B53] Zhang C. Xie G. Li S. Gai L. Qi Y. (2010). Spatial suitability distribution and ethanol production potential of energy crop Sweet Sorghum in China. Acta Ecol. Sin. 30, 4765–4770. doi: 10.20103/j.stxb.2010.17.027

[B54] Zhao D. Luan Y. Xia X. Shi W. Tang Y. Tao J. (2020). Lignin provides mechanical support to herbaceous peony (Paeonia lactiflora Pall.) stems. Hortic. Res. 7, 213. doi: 10.1038/s41438-020-00451-5, PMID: 33372177 PMC7769982

[B55] Zhong R. Morrison W. H. III Himmelsbach D. S. Poole F. L. Ye Z.-H. (2000). Essential role of caffeoyl coenzyme AO-methyltransferase in lignin biosynthesis in woody poplar plants. Plant Physiol. 124, 563–578. doi: 10.1104/pp.124.2.563, PMID: 11027707 PMC59163

[B56] Zhou L.-J. Xiao L.-T. Xue H.-W. (2017). Dynamic cytology and transcriptional regulation of rice lamina joint development. Plant Physiol. 174, 1728–1746. doi: 10.1104/pp.17.00413, PMID: 28500269 PMC5490912

[B57] Zou J. Wang Y. Li J. Zhu K. (2020). The grouping compatibility of excellent sorghum male sterile line 01-26A and its effect on plant height reduction and molecular mechanism. Chin. Agric. Sci. 53, 2814–2827. doi: 10.3864/j.issn.0578-1752.2020.14.006

