# Supplementary material for: Combined transcriptomic and metabolomic analysis of phenylpropanoid biosynthesis in the mechanism of leaf angle formation in Sorghum
Source: Front Plant Sci. 2025 Nov 10;16:1665475. doi: 10.3389/fpls.2025.1665475 (PMC12641002; doi:10.3389/fpls.2025.1665475)
Supplement: Supplementary file 1 [file Table1.docx]

Supplementary Material

# Supplementary Figures and Tables

## Supplementary Tables

**Supplementary Table 1.** qRT-PCR primer sequence.

| Primer name | Pathway name/Function | Sequence |
| --- | --- | --- |
| Sobic.007G076000F | Phenylpropanoid biosynthesis | AGTAAGGACGCCGAGGAGAT |
| Sobic.007G076000R | Phenylpropanoid biosynthesis | GATGAGATCAAACGGCGGGA |
| Sobic.005G011300F | Phenylpropanoid biosynthesis | GTTGCACCAACTTCAGAGCC |
| Sobic.005G011300R | Phenylpropanoid biosynthesis | TTGGTCAGAGTGTAGAAGCCC |
| Sobic.009G186600F | Phenylpropanoid biosynthesis | GGGACGCCGTCTACTTGAG |
| Sobic.009G186600R | Phenylpropanoid biosynthesis | TGTCCTTGGAGTTGAGCGAC |
| Sobic.003G342100F | Phenylpropanoid biosynthesis | GTTGCTTCTCCGGTCATCCT |
| Sobic.003G342100R | Phenylpropanoid biosynthesis | CTCAATGTCGGCCCAGGAG |
| Sobic.002G126600F | Phenylpropanoid biosynthesis | CTGGTGCCCCACATGAACC |
| Sobic.002G126600R | Phenylpropanoid biosynthesis | CGGAAGTCGTTGCCGTGT |
| Sobic.010G245500F | Phenylpropanoid biosynthesis | GTGCCAGTACAAGGTGTCGT |
| Sobic.010G245500R | Phenylpropanoid biosynthesis | GAGCTGGTCGAGGTTGAAGT |
| Sobic.001G112600F | internal standard | ATGGCTGACGCCGAGGATATCCA |
| Sobic.001G112600R | internal standard | GAGCCACACGGAGCTCGTTGTAG |

**Supplementary Table 2.** SEM analysis of the second leaf in the S1 for the WT and the *el1*.(Student’s t-test, *** p < 0.001, n = 5)

|  | **WT** | ***el1*** |
| --- | --- | --- |
| Number of abaxial cells | 80.60±1.82 | 59.80±2.17^****^ |
| Abaxial cell length（μm） | 52.81±2.77 | 63.93±3.56^***^ |
| Abaxial cell width（μm） | 14.81±1.89 | 24.18.±1.80^****^ |
| Number of adxial cells | 67.80±1.72 | 49.20±1.17^****^ |
| Adxial cell length（μm） | 54.89±3.89 | 72.91±3.12^****^ |
| Adxial cell width（μm） | 15.43±1.11 | 24.59±2.18^****^ |

**Supplementary Table 3.** Functional annotation of DEGs.

| **Gene_name** | **Pathway name** | **Function annotation** | | | **Regulated** | **log2FoldChange** |
| --- | --- | --- | --- | --- | --- | --- |
| Sobic.004G328700 | Phenylpropanoid biosynthesis | | Putrescine hydroxycinnamoyltransferase 1 | Down | | -1.049928769 |
| Sobic.002G205300 | Phenylpropanoid biosynthesis | | Putrescine hydroxycinnamoyltransferase 1 | Up | | 2.077076179 |
| Sobic.010G238600 | Phenylpropanoid biosynthesis | | Putrescine hydroxycinnamoyltransferase 1 | Up | | 1.182486783 |
| Sobic.003G082900 | Phenylpropanoid biosynthesis | | Putrescine hydroxycinnamoyltransferase 1 | Up | | 1.711156811 |
| Sobic.006G211900 | Phenylpropanoid biosynthesis | | Probable cinnamyl alcohol dehydrogenase 5 | Up | | 1.09308952 |
| Sobic.004G149600 | Phenylpropanoid biosynthesis | | Probable cinnamyl alcohol dehydrogenase 1 isoform X1 | Down | | -1.437520385 |
| Sobic.007G089900 | Phenylpropanoid biosynthesis | | Probable 4-coumarate--CoA ligase 1 | Down | | -1.119681744 |
| Sobic.004G105300 | Phenylpropanoid biosynthesis | | Peroxidase P7-like | Up | | 3.183597981 |
| Sobic.004G105100 | Phenylpropanoid biosynthesis | | Peroxidase P7 | Up | | 1.708734742 |
| Sobic.010G161800 | Phenylpropanoid biosynthesis | | Peroxidase P7 | Up | | 1.636224689 |
| Sobic.001G277000 | Phenylpropanoid biosynthesis | Peroxidase A2 | | Up | | 1.171201159 |
| Sobic.001G444500 | Phenylpropanoid biosynthesis | Peroxidase A2 | | Up | | 1.099111203 |
| Sobic.001G328100 | Phenylpropanoid biosynthesis | Peroxidase 70 | | Up | | 1.128943952 |
| Sobic.007G192300 | Phenylpropanoid biosynthesis | Peroxidase 51 | | Up | | 1.294154528 |
| Sobic.001G528100 | Phenylpropanoid biosynthesis | Peroxidase 5 | | Up | | 1.189931307 |
| Sobic.003G152200 | Phenylpropanoid biosynthesis | Peroxidase 5 | | Up | | 1.356073337 |
| Sobic.009G033300 | Phenylpropanoid biosynthesis | Peroxidase 5 | | Up | | 1.47574 |
| Sobic.010G245500 | Phenylpropanoid biosynthesis | Peroxidase 45 | | Down | | -1.452866127 |
| Sobic.005G011300 | Phenylpropanoid biosynthesis | Peroxidase 4 | | Up | | 2.606885898 |
| Sobic.008G010400 | Phenylpropanoid biosynthesis | Peroxidase 4 | | Up | | 1.014380412 |
| Sobic.001G189000 | Phenylpropanoid biosynthesis | Peroxidase 27 | | Up | | 3.503054965 |
| Sobic.002G416600 | Phenylpropanoid biosynthesis | Peroxidase 2 | | Up | | 3.493490151 |
| Sobic.002G234200 | Phenylpropanoid biosynthesis | Peroxidase 17 | | Up | | 1.29232119 |
| Sobic.009G186600 | Phenylpropanoid biosynthesis | Peroxidase 1 isoform X2 | | Up | | 1.821107556 |
| Sobic.003G152000 | Phenylpropanoid biosynthesis | Peroxidase 1 | | Up | | 1.265681573 |
| Sobic.003G151900 | Phenylpropanoid biosynthesis | Peroxidase 1 | | Up | | 1.469768015 |
| Sobic.003G082800 | Phenylpropanoid biosynthesis | Hydroxycinnamoyltransferase 4 isoform X1 | | Up | | 3.766112459 |
| Sobic.002G041900 | Phenylpropanoid biosynthesis | Hydroxycinnamoyltransferase 4 | | Up | | 1.555923199 |
| Sobic.010G066800 | Phenylpropanoid biosynthesis | Hydroxycinnamoyltransferase 4 | | Up | | 2.243772117 |
| Sobic.004G141200 | Phenylpropanoid biosynthesis | Cytochrome P450 CYP73A100 | | Up | | 2.718235166 |
| Sobic.003G342100 | Phenylpropanoid biosynthesis | Cinnamoyl-CoA reductase 1 | | Down | | -1.010825246 |
| Sobic.004G340200 | Phenylpropanoid biosynthesis | Cinnamoyl-CoA reductase 1 | | Up | | 5.104434201 |
| Sobic.006G277600 | Phenylpropanoid biosynthesis | Cationic peroxidase SPC4-like | | Up | | 2.475723614 |
| Sobic.005G000100 | Phenylpropanoid biosynthesis | Caffeoylshikimate esterase | | Up | | 1.025502062 |
| Sobic.003G203600 | Phenylpropanoid biosynthesis | Aldehyde dehydrogenase family 2 member C4 | | Up | | 2.180140315 |
| Sobic.010G178300 | Phenylpropanoid biosynthesis | Aldehyde dehydrogenase family 2 member C4 | | Up | | 2.023577426 |
| Sobic.002G009600 | Phenylpropanoid biosynthesis | 4-coumarate--CoA ligase-like 7 | | Down | | -1.402425128 |
| Sobic.001G189300 | Phenylpropanoid biosynthesis | 4-coumarate--CoA ligase-like 7 | | Up | | 1.625385954 |
| Sobic.002G389301 | Phenylpropanoid biosynthesis | 4-coumarate--CoA ligase-like 7 | | Up | | 1.865507098 |
| Sobic.002G126600 | Phenylpropanoid biosynthesis | Trans-cinnamate 4-monooxygenase | | Down | | -1.217777728 |
| Sobic.007G076000 | Phenylpropanoid biosynthesis | Probable cinnamyl alcohol dehydrogenase 5 | | Up | | 2.454461762 |
| Sobic.003G320800 | Phenylpropanoid biosynthesis | Peroxidase 1 | | Up | | 2.14396214 |
| Sobic.003G391600 | Phenylpropanoid biosynthesis | 4-coumarate--CoA ligase-like 5 | | Down | | -1.321521148 |

**Supplementary Table 4.** KEGG pathway of 19 DAMs.

| **Compounds** | **Type** | **Log2FC** | **Kegg map** |
| --- | --- | --- | --- |
| 3,5,7-Trihydroxyflavanone (Pinobanksin) | down | -2.21E+00 | ko00941 |
| 4-Hydroxyacetophenone | up | 1.21E+00 | ko01100 |
| Apigenin-7-O-(2''-apiosyl)glucoside (Apiin) | up | 1.03E+00 | ko00944 |
| Argininosuccinic acid | down | -1.06E+00 | ko00220, ko00250, ko01100, ko01110, ko01230 |
| Chrysoeriol5,7,4'-Trihydroxy-3'-Methoxyflavone | down | -1.01E+00 | ko00944 |
| Coniferyl alcohol | up | 1.81E+00 | ko00940, ko00999, ko01100, ko01110 |
| Cyclic 3',5'-Adenylic acid | up | 1.40E+00 | ko00230, ko01100 |
| Ergotamine | down | -1.77E+00 | ko01110 |
| Guanosine 3',5'-cyclic monophosphate | up | 1.83E+00 | ko00230, ko01100 |
| Homoeriodictyol | down | -1.01E+00 | ko00941 |
| L-Cystathionine | down | -1.71E+00 | ko00260, ko00270, ko01100, ko01110, ko01230, ko01240 |
| Liquiritigenin* | down | -1.12E+00 | ko00941, ko01100, ko01110 |
| N-Phenylacetylglycine | up | 3.93E+00 | ko00360 |
| Naringenin (5,7,4'-Trihydroxyflavanone)* | down | -2.00E+00 | ko00941, ko01100, ko01110 |
| Palmitoleic Acid* | up | 1.59E+00 | ko00061, ko01100 |
| Pimelic acid | up | 1.94E+00 | ko00780, ko01100, ko01240 |
| Pterostilbene | down | -1.01E+00 | ko00945, ko01110 |
| Pyridoxine | up | 1.96E+00 | ko00750, ko01100, ko01240 |
| Trans-5-O-(p-Coumaroyl)shikimate | up | 1.26E+00 | ko00940, ko00941, ko00945, ko01100, ko01110 |
