# Supplementary figures and images for: Combined transcriptomic and metabolomic analysis of phenylpropanoid biosynthesis in the mechanism of leaf angle formation in Sorghum
Source: Front Plant Sci. 2025 Nov 10;16:1665475. doi: 10.3389/fpls.2025.1665475 (PMC12641002; doi:10.3389/fpls.2025.1665475)

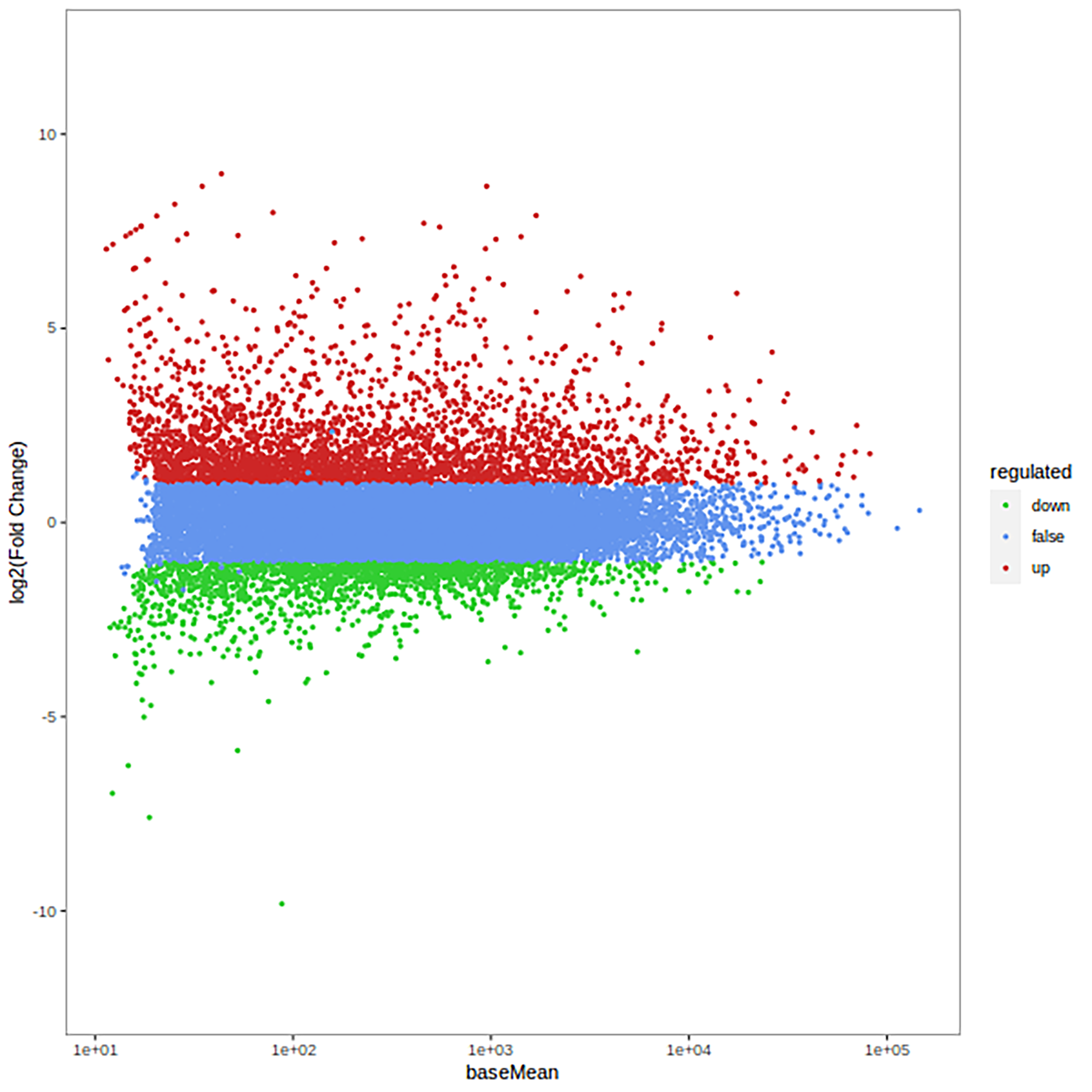

Supplement: Supplementary file 3 [file Image2.png]
